# Nine-Year Trends in Prevention of Thromboembolic Complications in Elderly Patients with Atrial Fibrillation Treated with NOACs

**DOI:** 10.3390/ijerph191911938

**Published:** 2022-09-21

**Authors:** Bernadetta Bielecka, Iwona Gorczyca-Głowacka, Beata Wożakowska-Kapłon

**Affiliations:** 1 1st Clinic of Cardiology and Electrotherapy, Swietokrzyskie Cardiology Centre, 25-736 Kielce, Poland; 2 Collegium Medicum, Jan Kochanowski University, 25-369 Kielce, Poland

**Keywords:** atrial fibrillation, non-vitamin K antagonist oral anticoagulants, apixaban, dabigatran, rivaroxaban

## Abstract

Background: Atrial fibrillation (AF) is the most common disease in elderly patients and thromboembolic complication prophylaxis significantly improves the prognosis in these patients. The study assessed the frequency of individual non-vitamin K antagonist oral anticoagulant (NOAC) use among patients ≥75 years and attempted to identify factors predisposing to their prescription. Methods: The data of patients with non-valvular AF hospitalized in the reference cardiology center between 2011 and 2019 were analyzed. Results: Out of 1443 analyzed patients, 329 (22.8%) received apixaban, 618 (42.8%) dabigatran, and 496 (34.4%) rivaroxaban. The entire population mean age was 82.3 ± 5 years, and 57.9% were females. Independent predictors of apixaban use were age, and bleeding history. Hospitalization for the implantation/reimplantation of a cardiac implantable electronic device (CIED) reduced the chance of apixaban use. Hypertension was a predictor of dabigatran prescription. The chance of using dabigatran decreased with age. Hypertension and bleeding history decreased the chance of rivaroxaban application. Conclusions: In hospitalized AF patients ≥75 years, dabigatran was the most frequently used NOAC. Age, comorbidities and bleeding risk determined the selection of individual NOACs.

## 1. Introduction

Atrial fibrillation (AF) is the most common disease in elderly patients and occurs in approximately 9 to 17% of people over 80 years of age [1,2]. Age is a risk factor not only for stroke, but also for bleeding, especially in patients with multiple factors which alter drug kinetics and the toxicity of standard doses of oral anticoagulants (OACs) [3]. Elderly patients (defined as those ≥ 75 years of age) usually have a low body mass index, changed muscle and fat composition, and age-related decline in kidney function [4]. In view of the projected increase in the incidence of AF in the world in the nearest future, there is an urgent need for effective stroke prevention strategies, especially in the elderly [5,6]. There is a clear need to optimize the use of anticoagulants in these patients, especially when they receive a full dose of the anticoagulant [7]. Long-term use of OACs in elderly patients with AF is recommended to reduce the risk of stroke. Until recently, the only oral anticoagulants available were vitamin K antagonists (VKAs) (e.g., warfarin). The new oral anticoagulants (NOACs) offer significant benefits and greater convenience to elderly patients as they have predictable pharmacological profiles, a rapid onset of action, a wide therapeutic window, no requirement for routine coagulation monitoring, and a smaller and better-defined number of food-drug interactions compared to warfarin [8]. Despite the benefits of anticoagulation shown in clinical trials, NOACs are underused in the elderly population [9]. Therefore, it is crucial to weigh the risks and benefits of anticoagulation strategies in this population [4]. 

The aim of our study was to assess the frequency of using individual non-vitamin K antagonist oral anticoagulants (NOACs) among elderly patients (≥75 years) and to try to identify factors that predispose to their recommendation. 

## 2. Materials and Methods

### 2.1. Study Group

The presented registry is a retrospective study of patients hospitalized in Świętokrzyskie Cardiology Center, which is the largest reference center in Świętokrzyskie Voivodeship, located in Kielce. It provides specialist medical care for the population of approximately 1,230,000 people in south-eastern Poland. Patients from both the Świętokrzyskie region and neighboring provinces are hospitalized there. Patients were included if they were at least 18 years of age and had a history of AF documented by electrocardiography or in their medical history. The study included patients with AF hospitalized between 2004 and 2019. Among all of the patients with AF hospitalized in the years 2004–2019, the first patient treated with NOAC was recorded in our registry in 2011. 

**Figure 1:**
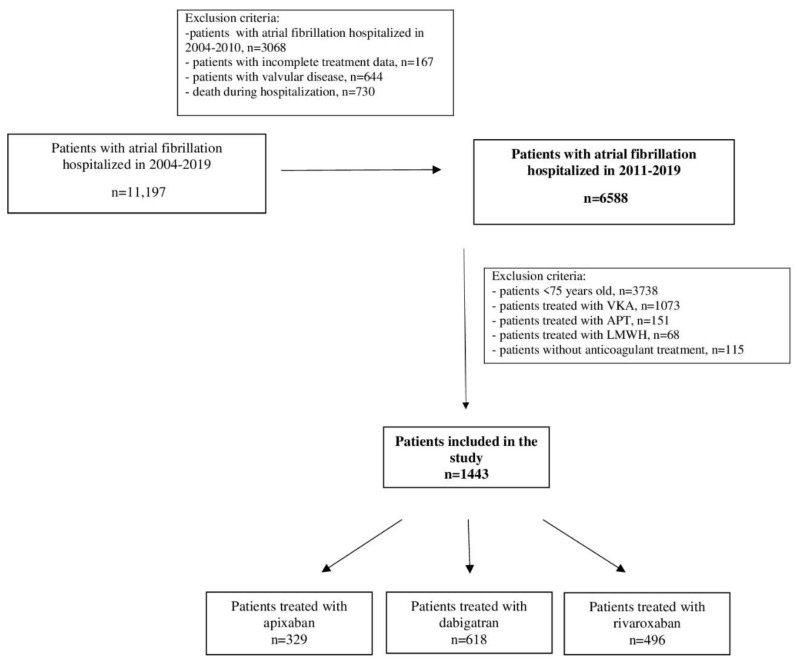
The flow chart of the study. Abbreviation: APT, antiplatelet drug; LMWH, low mass weight heparin; VKA, vitamin K antagonists.

The data of 6588 patients with AF hospitalized from 2011 to 2019 were analyzed. The registry excluded those ones with incomplete treatment data, patients with valvular disease, patients who died during hospitalization, patients under 75 years of age, and patients treated with VKAs, antiplatelet drugs (APT), low molecular weight heparin (LMWH) as well as patients without anticoagulant treatment (Figure 1). The presented study evaluated 1443 AF patients aged 75 and over treated with NOACs.

### 2.2. Assessed Parameters

Data were collected on gender, age, comorbidities, type of atrial fibrillation, laboratory and echocardiographic parameters, treatment method, and the reason for patients’ hospitalization. AF was diagnosed on the basis of the definition of the European Society of Cardiology, according to which arrhythmia can be identified using an electrocardiogram showing irregular atrial rhythm lasting longer than 30 s [10]. 

The glomerular filtration rate (GFR), which was used to assess the patients’ renal function, was calculated using the CKD-EPI (Chronic Kidney Disease Epidemiology Collaboration) equation. kLKThe study was approved by the Ethics Committee of the Świętokrzyska Medical Chamber in Kielce (Approval No. 12/2012). The Ethics Committee waived the requirement to obtain informed consent from patients. 

### 2.3. Assessment of the Thromboembolic Risk and Bleeding Risk 

Thromboembolic risk was assessed using the CHA_2_DS_2_-VASc score including congestive heart failure, hypertension, age ≥75 years, diabetes, stroke/transient ischemic attack (TIA)/thromboembolic event, vascular disease, age 65–74 years, female gender. 

The risk of bleeding was assessed on the basis of the HAS-BLED score including arterial hypertension, abnormal kidney/liver function, stroke, bleeding predisposition, unstable INR (international normalized ratio), the elderly (>65 years old), drug/alcohol consumption [11]. 

### 2.4. Prophylaxis of Thromboembolic Complications 

Patients treated with apixaban, dabigatran and rivaroxaban were compared. Edoxaban has been approved in Europe as a drug preventing thromboembolic complications in patients with AF, however, it is not available in Poland.

### 2.5. Statistical Analysis

In order to answer the research questions and test the hypotheses, statistical analyses were carried out using the IBM SPSS Statistics version 25 package. It was used to analyze basic descriptive statistics, chi square tests of independence, Fisher’s exact test, one-way ANOVA, Kruskal-Wallis tests and univariate logistic regression analyses. The classic threshold α = 0.05 was adopted as the level of statistical significance. In the first step of the analysis, the distributions of quantitative variables were checked. For this purpose, the basic descriptive statistics were calculated together with the Kolmogorov-Smirnow test examining the normality of the distribution. The differences among apixaban, dabigatran and rivaroxaban treated patients in terms of variables related to their general and clinical characteristics were tested sequentially. For this purpose, Kruskal-Wallis tests, chi-square tests of independence, Fisher’s exact test, and one-way ANOVA were performed. In order to deepen the analyses, a multivariate logistic regression analysis was performed. 

## 3. Results

### 3.1. Characteristics of the Study Group

Of the 1443 patients included in the analysis, 329 (22.8%) were treated with apixaban, 618 (42.8%) with dabigatran, and 496 (34.4%) with rivaroxaban. The reduced dose of the drug was administered to 920 (63.7%) patients, most frequently to patients taking dabigatran (78.8%) and least frequently to patients receiving apixaban (41.6%) (*p* < 0.001). 

The mean age of the entire population was 82.3 ± 5 years, and 57.9% of patients were women. Patients receiving apixaban were older (83.8 ± 5.3 years) compared to patients receiving dabigatran (81.7 ± 4.8 years) and rivaroxaban (81.9 ± 4.7 years) (*p* < 0.001). 

**Figure 2:**
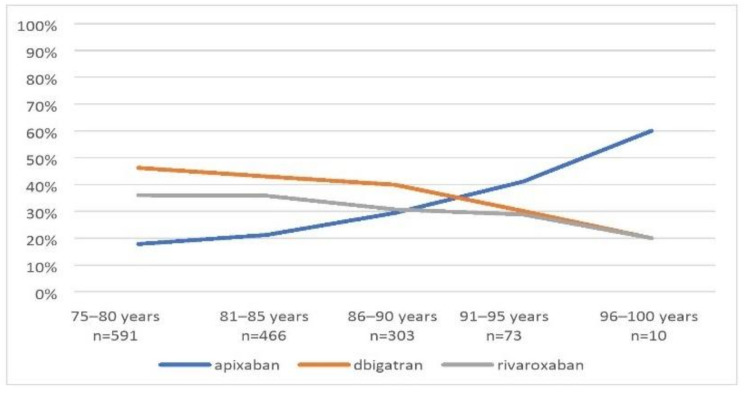
Division into individual NOACs depending on the age.

Among the oldest patients aged 96–100 years, apixaban accounted for 60% of prescribed NOACs, dabigatran 20%, rivaroxaban 20%. Among patients aged 75–80 years, the greatest number of patients received dabigatran—46.2%, and the lowest number of patients apixaban—17.8%. The distribution of individual NOACs in specific age groups is presented in Figure 2. 

**Figure 3:**
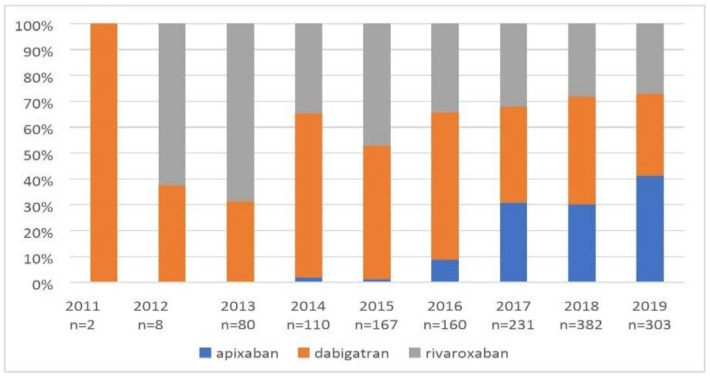
Temporal trends of anticoagulant therapy in all study patients treated with NOACs.

Figure 3 shows the use of NOACs in particular years.

**Figure 4:**
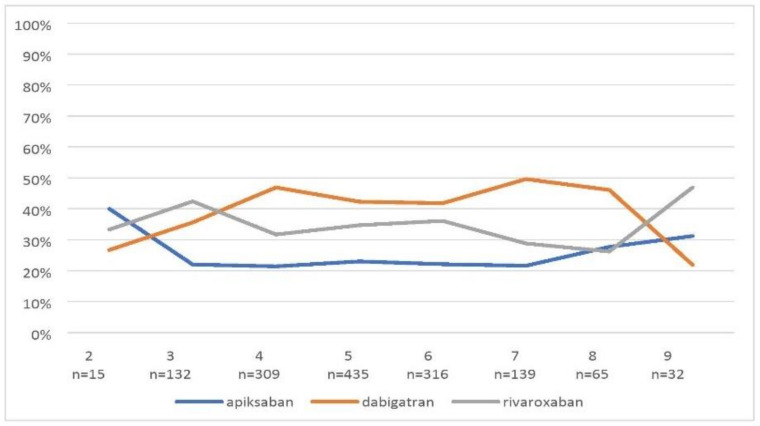
Division into individual NOACs depending on the CHA_2_DS_2_- VASc score.

The most common comorbidities in the study population were: arterial hypertension —1174 (81.3%) patients, heart failure—992 (68.7%) patients, vascular disease—693 (48%) patients and diabetes—439 (30.4%) patients. The mean CHA_2_DS_2_-VASc score was 5.2 ± 1.4 points. Figure 4 shows the division into individual NOACs depending on the score in the CHA_2_DS_2_-VASc score.

The mean HAS-BLED score was 2.2 ± 0.8 points. The result of ≥ 3 points was obtained by 466 (32.3%) people; moreover, higher values of the HAS-BLED score were observed among the respondents using dabigatran (34.6%) than with apixaban (26.7%) (*p* = 0.043). The most common reasons for hospitalization in the study group were as follows: heart failure—414 (28.7%) patients and cardiac implantable electronic device (CIED) implantation/reimplantation—301 (20.8%) patients. 

Among the patients receiving apixaban, heart failure was a significantly more frequent cause of hospitalization (36.5%) than among patients receiving dabigatran (27.7%) and rivaroxaban (24.8%) (*p* = 0.001). On the other hand, implantation and reimplantation of CIEDs as a reason for hospitalization are more common among patients using rivaroxaban (23.4%) than apixaban (15.5%) (*p* = 0.017). The characteristics of the study population are summarized in Table 1. 

### 3.2. Predictors of the Use of Individual NOACs

A series of univariate logistic regression analyses were performed for the use of apixaban, dabigatran and rivaroxaban. The variables included in the clinical characteristics of the group were included in the analyses. Numerous predictors of a specific NOAC selection were found in the univariate logistic regression analysis. (Table 2). 

In order to deepen the analyses, a multivariate logistic regression analysis was performed. The analyses were performed separately for the selection of the following drugs: apixaban, dabigatran and rivaroxaban. The model simultaneously included: age, gender, arterial hypertension, peripheral arterial disease (PAD) and history of bleeding, heart failure, ablation and CIED as the reason for hospitalization, and eGFR. The results of these analyses are included in Table 3.

The independent predictors of apixaban use were age (OR 1.08, 95% CI, 1.05–1.11; *p* < 0.001) and history of bleeding (OR 2.94, 95% CI, 1.71–5.06; *p* < 0.001). Hospitalization for CIED decreased the chance of apixaban use (OR 0.63, 95% Cl, 0.44–0.90; *p* = 0.011). Hypertension was a predictor of the use of dabigatran (OR 1.56, 95% Cl, 1.18–2.06; *p* = 0.022). With age, the chance of using dabigatran decreased (OR 0.96, 95% Cl, 0.94–0.98; *p* < 0.001). Hypertension (OR 0.74, 95% Cl, 0.56–0.98; *p* = 0.034) and history of bleeding (OR 0.50, 95% Cl, 0.26–0.95; *p* = 0.034) decreased the chance of receiving rivaroxaban.

## 4. Discussion

The present study has several major findings. Firstly, the most frequently chosen non-vitamin K antagonist oral anticoagulant (NOAC) in elderly patients was dabigatran. Secondly, age, comorbidities, and risk of bleeding complications were predictors for the selection of individual NOACs.

The use of individual NOACs in our population varied depending on the patient profile. The choice of NOACs was also influenced by the patient’s comorbidities, the risk of bleeding and the current safety studies available. Physicians prescribing a given drug were guided by evidence-based medicine and clinical experience. Our study population consists of elderly patients at higher risk of complications and potential risks. After approval of apixaban, which can also be used in advanced kidney disease, it was more likely to be prescribed to older patients. 

In the presented registry, the most frequently chosen drug was dabigatran. It was the first NOAC registered in the world. Our study was conducted between 2011 and 2019, hence probably the greatest popularity of dabigatran. Similarly, in the Adeboyeje study [12] conducted in 2010–2015, where dabigatran was in the first place in terms of prevalence among NOACs, and rivaroxaban was in the second place. On the other hand, the data from the PINNACLE NCDR registry showed that rivaroxaban was used more often than dabigatran and apixaban [13]. The highest percentage of patients treated with apixaban was reported in the Norwegian patient registry [14]. In Poland, apixaban has been approved as the third NOAC, which is why it is the lowest in our registry in terms of quantity. It should be said that despite the lowest popularity of apixaban in our study, the number of prescriptions is steadily increasing, and it is likely to overtake the number of prescriptions in relation to dabigatran and rivaroxaban in the future. 

In our registry, it was also observed that a large percentage of patients received the reduced dose of the drug; it was as much as 63.7% of people, which was the most among patients using dabigatran. This reduction in NOAC dose is mainly due to limitations of renal functions that occur with increasing age. The dose reduction was also the result of a triple anticoagulant treatment in patients with acute coronary syndromes (ACS) and after percutaneous coronary intervention (PCI). Drug dosing depends primarily on estimated creatinine clearance, mainly calculated using the Cockroft-Gault formula. The medical records in our registry were based on the assessment of kidney function in accordance with MDRD (Modification of Diet in Renal Disease). Although the methods are similar, there are differences between them that may have been reflected in the use of the correct dose of the drug. However, we cannot exclude a dose reduction based on other features, such as history of bleeding, peptic ulcer disease, low blood counts, and the preferences of physicians directly assessing the patient’s clinical condition. 

In our study, the mean eGFR value for the study population was only 49.9 mL/min/1.73 m^2^. In the study by Rutheford et al. [15] also the highest number of patients ≥75 years using dabigatran received a reduced dose. This is probably due to the fact that dabigatran was the first and best-studied NOAC available, and therefore doctors had fewer concerns when prescribing it to the oldest and most heavily burdened group of patients requiring a lower dose of the drug. We assessed elderly patients with multiple comorbidities who had usually a low body mass index, which explains the above conclusions.

One of the conclusions of the study is that the older the patients are, the higher the chance of prescribing apixaban. A study by Zeitouni et al. confirms effectiveness and safety also of a reduced dose, which is especially important for seniors [16]. Elderly patients are at increased risk of stroke and systemic embolism, as well as bleeding, hence the need for effective anticoagulation. In the OBIT-AF II study, advanced age predisposed the choice of apixaban compared to rivaroxaban [17]. This is confirmed by the study by Rutheford et al. [14], in which patients using dabigatran were younger and had fewer comorbidities. Similarly, in the study by Yao et al. patients using dabigatran were younger than those using apixaban and rivaroxaban [18]. 

In our study, hospitalization for the implantation/reimplantation of a cardiac implantable electronic device (CIED) decreased the chance of using apixaban compared to dabigatran and rivaroxaban. It is probably related to the insufficient follow-up and results regarding this drug. In the registry of Black-Maier et al. [19], people undergoing CIED implantation who used NOACs were younger and had fewer comorbidities, compared to patients using warfarin, which proves the need for caution when using these drugs. Apixaban, on the other hand, is more willingly prescribed to elderly people with multiple comorbidities. In the ESS-PREDI study, in patients who underwent CIED implantation/surgical revision as a part of long-lasting anticoagulant therapy [20], minor pocket hematomas and bleeding complications were significantly less frequent in patients treated with NOACs compared to those treated with vitamin K antagonists (VKAs) and antiplatelet drugs. In the study by Chuan-Tsai et al., where NOACs were used in patients with AF and high thromboembolic risk during CIED implantation, no cases of major bleeding were reported; moreover, no periprocedural mortality or strokes were observed [21]. In the retrospective study comparing the occurrence of bleeding and thromboembolic complications after CIED implantation in 176 patients treated with rivaroxaban or dabigatran, no differences in 30-day bleeding complications between groups were found, but it should be emphasized that only discontinued therapy was analyzed [22]. Therefore, more research is needed to prove the direct effectiveness of NOACs in patients undergoing implantation and reimplantation of implantable devices. 

In our registry, arterial hypertension predisposed patients to the choice of dabigatran. In the Canadian registry, among patients over 65 years of age in long-term care facilities, patients with arterial hypertension were more likely to be prescribed NOACs [23]. In patients with AF, arterial hypertension is not only a risk factor for stroke, but it is also associated with an increased risk of bleeding in people receiving anticoagulants [24,25]. Ishii et al. write about the correlation between the occurrence of bleeding and arterial hypertension in patients with AF, and thus using anticoagulants [26]. Due to the higher risk of bleeding among patients taking rivaroxaban, hypertension was a factor which probably decreased the chances of prescribing this drug, and dabigatran was the preferred and recommended choice. On the other hand, in another study, in a subgroup of Japanese patients in the ROCKET-AF study [27] which compared patients with AF and hypertension taking warfarin or rivaroxaban, it was observed that the safety and efficacy profile of rivaroxaban was similar to that of warfarin, regardless of baseline hypertension. Diener et al. write that when comparing NOACs, no differences were found in terms of safety or efficacy in patients with AF and arterial hypertension [28]. The results of the research depend on the selection of the test group and the time of the research.

History of bleeding was a factor which predisposed the choice of apixaban but decreased the chance of rivaroxaban prescription.

In the ARISTOTLE study [29], the use of apixaban in the elderly reduced the incidence of stroke or systemic embolism by 29% and major bleeding by 36%. In the meta-analysis by Malik et al. [30], apixaban was the only oral anticoagulant that significantly reduced all 3 results i.e., systemic embolism, major bleeding and intracranial hemorrhage compared to warfarin. In the meta-analysis by Sharm et al. comparing NOACs and warfarin in the group of patients >75 years of age, dabigatran in particular was associated with a higher risk of gastrointestinal bleeding compared to VKAs [31], which suggests its greater safety in younger patients. Analyses of the RE-LY data suggested a lower risk of major bleeding in patients <75 years of age, but a trend to a higher risk in patients ≥75 years of age [32]. Our analysis is based on patients at high risk of thromboembolism, and includes patients over 75 years of age, which is consistent with the results of the cited studies. In contrast, Noseworthy et al. [33] found no significant differences in efficacy between NOACs, and both dabigatran and apixaban were associated with a significantly lower risk of bleeding compared to rivaroxaban. This registry, created in the United States between 2010 and 2015, shows that rivaroxaban may be associated with an increased risk of major bleeding and intracranial bleeding. Graham et al. [34] conducted a retrospective cohort study of new users including 118,891 patients with non-valvular AF (NVAF), which showed that the standard dose of rivaroxaban was associated with more frequent cases of serious bleeding events than the standard dose of dabigatran, which is also consistent with our research. 

Concerns related to bleeding are still the main reason for refraining from prescribing new oral anticoagulants, but the studies mentioned partially resolve these doubts. Depending on a given clinical situation, administration of a specific NOAC is preferable, therefore the factors that predisposed to the use of a specific drug were investigated. 

## 5. Study Strengths and Limitations

Our study was conducted on the basis of documentation collected in one centre, a reference clinic in the voivodeship city. The registry includes patients from a vast geographical region, it contains a significant number of people both from Świętokrzyskie Voivodeship and the surrounding voivodeships. It includes only patients at high thromboembolic risk who therefore need anticoagulant therapy. Due to the long observation period, it is not possible to clearly determine the starting point for the application of a specific NOAC, as they were introduced in Poland at different times. Nevertheless, patients were treated according to the latest reliable medical knowledge in correlation to the successively published guidelines for atrial fibrillation. This provides a consistent and clear picture of the management of patients at high thromboembolic risk, as well as a complete overview of medical management and practice.

## 6. Conclusions

The presented study demonstrates an up-to-date picture of the use of NOACs in elderly patients with AF. This study extends the knowledge of contemporary AF management and demonstrates good implementation of clinical guidelines for stroke prevention. Factors influencing the selection of NOACs were identified: age, comorbidities and risk of bleeding. In the elderly population, it is particularly important to individualize anticoagulant therapy due to the increased risk of thromboembolic and hemorrhagic complications.

## Figures and Tables

**Table 1:** Clinical characteristics of patients treated with apixaban, dabigatran and rivaroxaban. Results of the laboratory test and echocardiographic examinations of patients treated with apixaban, dabigatran and rivaroxaban.

Clinical Characteristic	All NOAC n = 1443	Apixaban n = 329	Dabigatran n = 618	Rivaroxaban n = 496	*p*
**Age**					
Mean (SD), years	82.3 (5)	83.8 (5.3)	81.7 (4.8)	81.9 (4.8)	**<0.001**
Median (IQR)	82 (8)	84 (8) _a_	81 (7) _b_	82 (7) _b_	
**Gender**					
Female, n (%)	836 (57.9)	193 (58.7)	338 (54.7)	305 (61.5)	0.070
**Type of atrial fibrillation n(%)**					
Paroxysmal	657 (45.5)	148 (45)	295 (47.7)	214 (43.1)	0.303
Persistent	154 (10.7)	35 (10.6)	57 (9.2)	62 (12.5)	0.212
Permanent	632 (43.8)	146 (44.4)	266 (43)	220 (44.4)	0.882
Non-permanent	811 (56.2)	183 (55.6)	352 (57)	276 (55.6)	0.882
**Medical history n(%)**					
Hypertension	1174 (81.3)	259 (78.7) _ab_	524 (84.8) _b_	391 (78.8) _a_	**0.015**
Heart failure	992 (68.7)	237 (72)	419 (67.8)	336 (67.7)	0.342
Vascular disease	693 (48)	164 (49.8)	301 (48.7)	228 (46)	0.498
Previous myocardial infarction	351 (24.3)	93 (28.3)	149 (24.1)	109 (22)	0.118
PAD	159 (11)	45 (13.7)	71 (11.5)	43 (8.7)	0.071
Previous stroke/TIA/peripheral embolism	244 (16.9)	55 (16.7)	111 (18)	78 (15.7)	0.610
Diabetes mellitus	439 (30.4)	106 (32.2)	183 (29.6)	150 (30.2)	0.704
Any previous bleeding	59 (4.1)	27 (8.2) _a_	20 (3.2) _b_	12 (2.4) _b_	**<0.001**
Ulcer	36 (2.5)	4 (1.2)	15 (2.4)	17 (3.4)	0.136
Malignancy	81 (5.6)	22 (6.7)	31 (5)	28 (5.6)	0.568
**Thromboembolic risk**					
**CHA_2_DS_2_-VAS_C_ score**					
Mean (SD)	5.2 (1.4)	5.3 (1.5)	5.2 (1.4)	5.2 (1.4)	0.644
**Bleeding risk**					
**HAS-BLED score**					
Mean (SD)	2.2 (0.8)	2.2 (0.7)	2.3 (0.7)	2.2 (0.8)	**0.026**
Median (IQR)	2 (1)	2 (1) _a_	2 (1) _b_	2 (1) _ab_	
≥3, n (%)	466 (32.3)	88 (26.7) _a_	214 (34.6) _b_	164 (33.1) _ab_	**0.043**
**Reason for hospitalisation, n(%)**					
Electrical cardioversion	96 (6.6)	18 (5.5)	42 (6.8)	36 (7.3)	0.591
Planned coronarography/PCI/ACS	115 (8)	31 (9.4)	44 (7.1)	40 (8.1)	0.458
Heart failure	414 (28.7)	120 (36.5) _a_	171 (27.7) _b_	123 (24.8) _b_	**0.001**
Ablation	17 (1.2)	2 (0.6)	5 (0.8)	10 (2)	0.122
CIED	301 (20.8)	51 (15.5) _a_	133 (21.5) _ab_	116 (23.4) _b_	**0.017**
AF attack	157 (10.9)	32 (9.7)	78 (12.6)	47 (9.5)	0.183
Other	343 (23.8)	75 (22.8)	145 (23.5)	123 (24.8)	0.781
**Laboratory tests**					
**Haemoglobin**					
Mean (SD), g/dl	12.9 (3.3) n = 1418	12.5 (1.7) n = 319	13.1 (4.7) n = 609	12.8 (1.6) n = 490	**<0.001**
Median (IQR)	12.8 (2.1)	12.4 (2.2) _a_	12.9 (2.1) _b_	12.9 (2) _b_	
**Platelet**					
Mean (SD), K/uL	209.9 (75.3) n = 1410	207.6 (79.5) n = 318	207.2 (72.6) n = 604	214.6 (75.7) n = 488	0.113
Median (IQR)	198 (78)	197.5 (93.8)	194 (72)	202.5 (76)	
**eGFR**					
Mean (SD), mL/min/1.73 m^2^	49.9 (14.8) n = 1438	45.8 (17.3) n = 327	52.3 (12.6) n = 616	49.7 (14.9) n = 495	**<0.001**
Median (IQR)	49.3 (19.3)	43 (24.8) _a_	51.2 (17.2) _b_	49.2 (19.6) _c_	
<60 mL/min/1.73 m^2^, n (%)	1107 (77) n = 1438	264 (80.7) N = 327	458 (74.4) n = 616	385 (77.8) n = 495	0.088
**Echocardiographic findings**					
**Ejection fraction, mm**					
Mean (SD)	49.2 (20.3) n = 1185	47 (12.5) n = 269	50.3 (27.4) n = 516	49.4 (12) n = 400	**0.021**
Median (IQR)	50 (18)	50 (17) _a_	52 (17) _b_	50 (15) _ab_	
**Left ventricular systolic diameter, mm**					
Mean (SD)	36.3 (9.5) n = 1160	36.1 (9.2) n = 263	37 (10) n = 509	35.7 (8.8) n = 388	0.352
Median (IQR)	35 (11)	35 (12)	35 (12)	34 (10)	
**Left ventricular diastolic diameter, mm**					
Mean (SD)	50.2 (8) n = 1168	49.2 (8.5) n = 268	50.9 (8) n = 511	49.9 (7.6) n = 389	**0.016**
Median (IQR)	49 (10)	48.5 (12) _a_	50 (11) _b_	49 (10) _ab_	
Reduced dose, n (%)	920 (63.7)	137 (41.6) _a_	487 (78.8) _b_	296 (59.7) _c_	**<0.001**
Antiplatelet with NOAC, n (%)	104 (7.2)	31 (9.4)	39 (6.3)	34 (6.9)	0.197

Different letters between the scores indicate significant differences at the level *p* < 0.05; *p* < 0.05 corresponds to the comparison between the 2 drugs with the same letter. Data are presented as number (percentage) or mean (standard deviation) (SD)) or median (interquartile range) (IQR). Abbreviations: ACS, acute coronary syndromes; AF, atrial fibrillation; CI, confidence interval; CIED, cardiac implantable electronic device; eGFR, estimated Glomerular Filtration Rate; PAD, peripheral artery disease; PCI, percutaneous coronary interventions; TIA, transient ischaemic attack.

**Table 2:** Results of univariate regression analyses for the use of apixaban, dabigatran and rivaroxaban, respectively.

	Apixaban			Dabigatran			Rivaroxaban		
Factors	OR	95% CI	*p*	OR	95% CI	*p*	OR	95% CI	*p*
Age	1.08	1.06–1.11	**<0.001**	0.96	0.94–0.98	**<0.001**	0.98	0.96–1.00	0.078
Gender	1.04	0.81–1.33	0.761	0.79	0.64–0.98	**0.031**	1.25	1.00–1.56	**0.048**
**Type of atrial fibrillation**									
Paroxysmal	0.97	0.76–1.24	0.821	1.17	0.95–1.44	0.146	0.86	0.69–1.08	0.188
Persistent	1.00	0.67–1.48	0.982	0.76	0.54–1.08	0.124	1.33	0.94–1.87	0.104
Permanent	1.03	0.81–1.32	0.810	0.95	0.77–1.17	0.617	1.04	0.83–1.29	0.758
Non-permanent	0.97	0.76–1.24	0.810	1.06	0.86–1.30	0.617	0.97	0.78–1.20	0.758
**Medical history**									
Hypertension	0.81	0.59–1.09	0.805	1.50	1.14–1.98	**0.004**	0.78	0.59–1.03	0.075
Heart failure	1.23	0.93–1.61	0.143	0.93	0.74–1.16	0.502	0.93	0.74–1.18	0.552
Vascular disease	1.10	0.86–1.41	0.451	1.05	0.85–1.29	0.654	0.88	0.71–1.10	0.258
Previous myocardial infarction	1.31	0.99–1.73	0.058	0.98	0.77–1.25	0.870	0.82	0.63–1.06	0.133
PAD	1.39	0.96–2.01	0.081	1.09	0.78–1.52	0.622	0.68	0.47–0.98	**0.040**
Previous stroke/TIA/peripheral embolism	0.98	0.71–1.37	0.916	1.14	0.86–1.50	0.356	0.58	0.26–1.29	0.183
Diabetes mellitus	1.16	0.86–1.45	0.420	0.94	0.75–1.17	0.562	0.99	0.78–1.25	0.914
Any previous bleeding	3.03	1.78–5.13	**<0.001**	0.67	0.39–1.17	0.159	0.48	0.25–0.90	**0.023**
Ulcer	0.42	0.15–1.19	0.101	0.95	0.49–1.86	0.887	1.73	0.89–3.37	0.104
Malignancy	1.28	0.77–2.13	0.337	0.82	0.52–1.30	0.394	1.01	0.63–1.62	0.970
**Thromboembolic risk**									
CHA2DS2-VASC score	1.03	0.94–1.12	0.539	1.01	0.94–1.09	0.736	0.97	0.89–1.04	0.372
**Bleeding risk**									
HAS-BLED score	0.91	0.78–1.08	0.283	1.21	1.05–1.39	**0.008**	0.87	0.76–1.01	0.068
≥3, n (%)	0.71	0.54–0.94	**0.015**	1.20	0.96–1.50	0.101	1.06	0.84–1.33	0.650
**Reason for hospitalisation**									
Electrical cardioversion	0.77	0.45–1.30	0.329	1.04	0.69–1.58	0.850	1.16	0.75–1.78	0.505
Planned coronarography/PCI/ACS	1.28	0.83–1.96	0.269	0.81	0.55–1.20	0.303	1.02	0.68–1.52	0.923
Heart failure	1.60	1.23–2.08	**<0.001**	0.92	0.73–1.16	0.458	0.74	0.58–0.95	**0.018**
Ablation	0.45	0.10–1.97	0.288	0.55	0.19–1.58	0.268	2.76	1.05–7.30	**0.040**
CIED	0.63	0.46–0.88	**0.007**	1.07	0.83–1.39	0.592	1.28	0.99–1.66	0.065
AF attack	0.85	0.57–1.28	0.445	1.36	0.98–1.90	0.067	0.80	0.56–1.14	0.216
**Laboratory test**									
Haemoglobin	0.86	0.79–0.93	**<0.001**	1.10	1.03–1.17	**0.006**	0.99	0.96–1.03	0.749
Platelet	1.00	1.00–1.00	0.999	1.00	1.00–1.00	0.249	1.00	1.00–1.00	0.085
eGFR	0.97	0.97–0.98	**<0.001**	1.10	1.01–1.03	**<0.001**	1.00	0.99–1.01	0.724
eGFR < 60 mL/min/1.73 m^2^	1.31	0.96–1.77	0.085	0.78	0.61–0.99	**0.043**	1.08	0.83–1.40	0.556
**Echocardiographic findings**									
Ejection fraction	0.99	0.98–1.00	**0.007**	1.01	1.00–1.01	0.166	1.00	1.00–1.01	0.857
Left ventricular systolic diameter	1.00	0.98–1.01	0.633	1.01	1.00–1.03	**0.048**	0.99	0.98–1.00	0.097
Left ventricular diastolic diameter	0.98	0.96–1.00	**0.015**	1.02	1.01–1.04	**0.005**	0.99	0.98–1.01	0.440
**Reduced dose**	0.30	0.23–0.39	**<0.001**	3.37	2.66–4.26	**<0.001**	0.77	0.61–0.96	**0.020**
**Antiplatelet with NOAC**	1.48	0.96–2.30	0.079	0.79	0.52–1.19	0.255	0.92	0.60–1.41	0.708

Abbreviations: ACS, acute coronary syndromes; AF, atrial fibrillation; CI, confidence interval; CIED, cardiac implantable electronic device; eGFR, estimated Glomerular Filtration Rate; NOAC, novel oral anticoagulants; PAD, peripheral artery disease; PCI, percutaneous coronary interventions; TIA, transient ischaemic attack; OR, odds ratio.

**Table 3:** Predictors of drug selection in the multivariate logistic regression analysis.

	Apixaban _1_			Dabigatran _2_			Rivaroxaban _3_		
Factors	OR	95% CI	*p*	OR	95% CI	*p*	OR	95% CI	*p*
Age	1.08	1.05–1.11	**<0.001**	0.96	0.94–0.98	**<0.001**	0.98	0.96–1.00	0.094
Gender	0.99	0.76–1.29	0.916	0.82	0.65–1.02	0.071	1.25	0.99–1.58	0.058
Hypertension	0.80	0.58–1.10	0.168	1.56	1.18–2.06	**0.022**	0.74	0.56–0.98	**0.034**
PAD	1.20	0.82–1.76	0.351	1.17	0.83–1.64	0.373	0.72	0.49–1.04	0.081
Any previous bleeding	2.94	1.71–5.06	**<0.001**	0.68	0.39–1.18	0.167	0.50	0.26–0.95	**0.034**
Heart failure	1.28	0.96–1.71	0.093	0.98	0.76–1.26	0.873	0.83	0.63–1.08	0.157
Ablation	0.59	0.13–2.64	0.491	0.52	0.18–1.51	0.228	2.39	0.89–6.41	0.084
CIED	0.63	0.44–0.90	**0.011**	1.11	0.84–1.46	0.470	1.25	0.94–1.66	0.126
eGFR < 60 mL/min/1.73 m^2^	1.20	0.87–1.66	0.266	0.83	0.65–1.08	0.164	1.07	0.82–1.41	0.604

_1_: χ2(9) = 74.39; *p* < 0.001; Nagelkerke *R^2^* = 0.08; _2_: χ2(9) = 33.98; *p* < 0.001; Nagelkerke *R^2^* = 0.03, _3_: χ2(9) = 30.51; *p* < 0.001; Nagelkerke *R^2^* = 0.03. Abbreviations: CIED, cardiac implantable electronic device; eGFR, estimated Glomerular Filtration Rate; PAD, peripheral artery disease; OR, odds ratio.

## Data Availability

Data is available on request for the first author.
